# Survey of the Influence of the Width of Urban Branch Roads on the Meeting of Two-Way Vehicle Flows

**DOI:** 10.1371/journal.pone.0149188

**Published:** 2016-02-16

**Authors:** Qun Chen, Yunan Zhao, Shuangli Pan, Yan Wang

**Affiliations:** 1 School of Traffic and Transportation Engineering, Central South University, Changsha, China; 2 School of Public Administration, Central South University, Changsha, China; Beihang University, CHINA

## Abstract

Branch roads, which are densely distributed in cities, allow for the flow of local traffic and provide connections between the city and outlying areas. Branch roads are typically narrow, and two-way traffic flows on branch roads are thus affected when vehicles traveling in opposite directions meet. This study investigates the changes in the velocities of vehicles when they meet on two-way branch roads. Various widths of branch roads were selected, and their influence on traffic flows was investigated via a video survey. The results show that, depending on the average vehicle velocity, branch roads require different widths to prevent a large decrease in velocity when vehicles meet. When the velocity on a branch road is not high (e.g., the average velocity without meeting is approximately 6 m/s), appropriately increasing the road width will notably increase the meeting velocity. However, when the velocity is high (e.g., the average velocity without meeting is greater than 10 m/s), there is a large decrease in velocity when meeting even if the road surface is wide (6.5 m). This study provides a basis for selecting the width of urban branch roads and the simulation of bidirectional traffic on such roads.

## Introduction

Urban branch roads connect neighborhoods with major arteries and accommodate local traffic. The characteristics of branch roads are as follows. (1) The number of branch roads is large. Urban branch roads are capillaries of the urban transportation networks that connect the neighborhood with the surrounding region and provide connections within the neighborhood. (2) The width of a branch road is typically narrow. On both sides of branch roads, buildings are typically densely distributed, so the road cannot be particularly wide. In addition, because the number of branch roads is large, a considerable amount of land will be occupied by road surfaces if the widths of branch roads are large. Further, because the number of branch roads is large, the traffic volume is scattered, and the traffic volume on a branch road is typically not large; thus, a wide road surface will be a waste of resources. Moreover, urban branch roads typically provide a service for the livelihoods of the residents; thus, they cannot be overly wide. In many cases, they are two-lane roads allowing for two-way vehicle flows, with the lane width narrower than those of arterial roads. (3) The traffic volume on branch roads is typically small. Because branch roads are widely distributed in cities, the traffic volume encountered on a branch road is typically not large.

Because branch roads are narrow, they typically have no clear separating structures and pavement markings or only simple line markings in the center of the road. As a result, the bidirectional flow on branch roads causes vehicles to decelerate considerably when meeting. These changes in velocity can cause serious congestion on branch roads. The road width prominently affects vehicle velocities. This study investigates the influence of the road width on bidirectional traffic flows via a field survey, thereby providing design guidelines for the widths of branch roads to avoid waste and reduce delays caused by meetings of opposing vehicles. Moreover, parameters are provided for micro-scale simulations of traffic on branch roads through an analysis of variations in the velocities of two-way traffic flows on branch roads of various widths. Currently, simulations of such branch roads assume two independent flows without interactions [1–9], which does not correspond to the actual circumstances. In actual situations, vehicles traveling in opposite directions decelerate when they meet, which causes delays for the vehicles. However, this delay is related to the road width, and different road widths result in different meeting velocities and driving behaviors. This study investigates the driving behaviors for various road widths and provides parameters for traffic simulations of branch roads.

Previous studies have examined the influence of road width on vehicle speeds, road capacity [10–20] and safety [21, 22]; however, no study has been reported to investigate the effect of road width on two-way vehicles’ meeting. There have been many studies on traffic simulation models such as kinematic wave models [1, 2], cellular automata (CA) simulation models [6, 8, 9], car-following models [7, 23, 24] and vehicle cruise control models [25–27]. However, few studies have considered the interaction of two-way traffic on branch roads. In one study, Popkov and Peschel [28] transformed this problem into two unidirectional traffic flows with modified parameters. Lee et al. [29, 30] proposed a two-way traffic flow model based on the asymmetric simple exclusion process (ASEP), in which the approaching car in the opposite lane reduces the hopping rate of the subject vehicle. Although previous studies acknowledged that opposing traffic affects traffic flows and constructed traffic flow models for interacting bidirectional traffic, the dependency of vehicle velocities on the road width in actual cases was not investigated. This study provides parameters for interacting two-way traffic flow models.

The remainder of the paper is organized as follows. In Section 2, we describe the survey methodology, Section 3 presents the survey results, Section 4 discusses the results of a statistical analysis and draws comparisons with existing criteria, and conclusions are then given in Section 4.

## Methods

The city in which the survey was conducted was Changsha, China, and the surveyed roads were two-way, two-lane branch roads. In the survey, the road width of each selected road was recorded, and video samples of the meetings of opposing vehicles were obtained.

### Road sections

**Fig 1:**
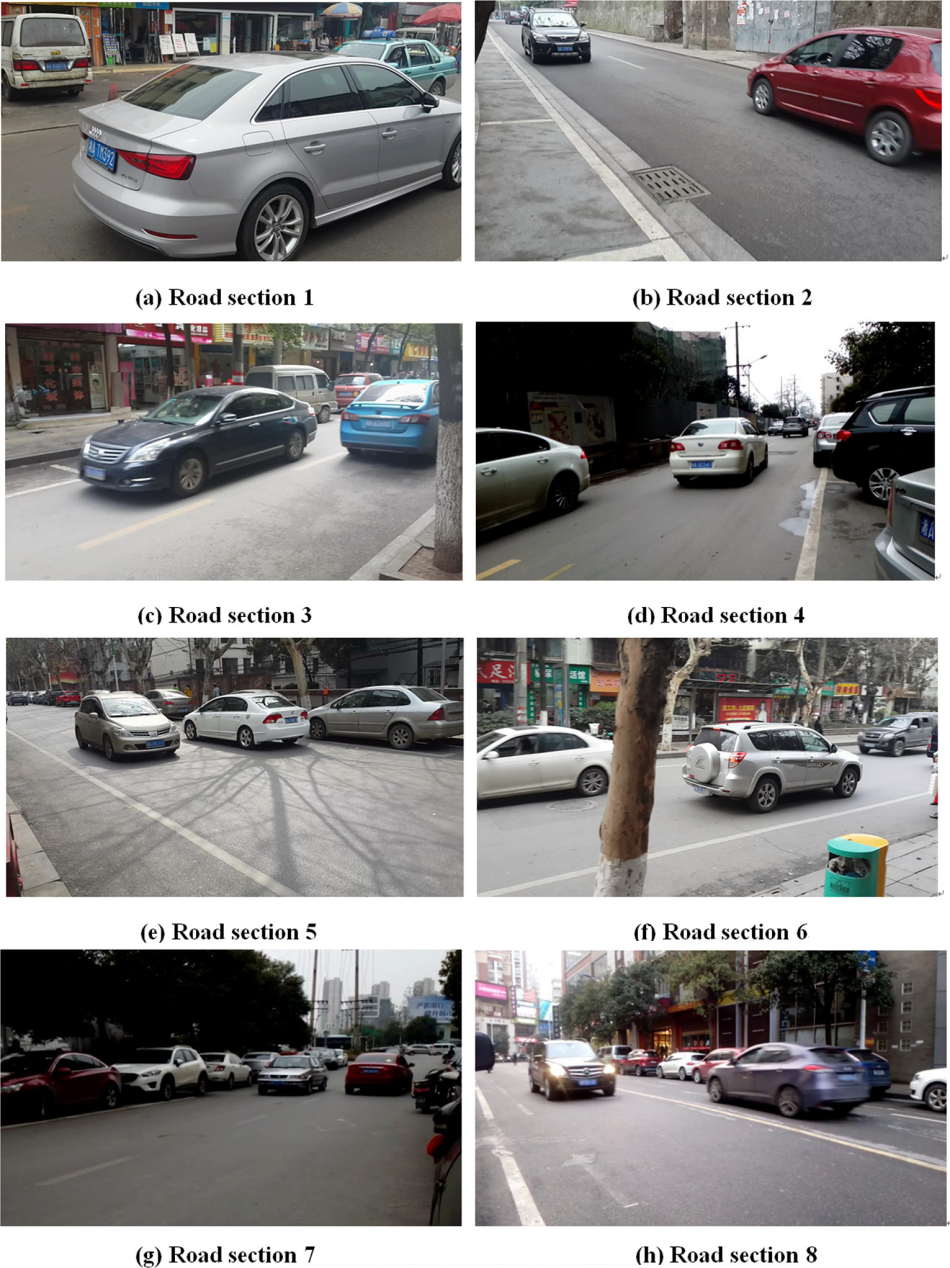
Photographs of the surveyed road sections.

Table 1 lists the widths of the selected road sections, and Fig 1A–1H are actual photographs of the survey road sections. In the survey, the samples that may have been affected by other factors such as pedestrians and bicycles were discarded. In addition, only road sections with low traffic volumes were selected for the survey to eliminate the influence of congestion due to heavy traffic, and therefore the vehicle speeds were primarily related to the road width.

**Table 1:** Widths of the road sections.

Road section	1	2	3	4	5	6	7	8
Width (m)	4.56	4.82	5.02	5.30	5.62	6.5	6.6	7.6
Remarks			Parking spaces exist on one side	Parking spaces exist on one side	Parking spaces exist on both sides	Parking spaces exist on both sides	Parking spaces exist on both sides	Parking spaces exist on both sides

### Average velocity without vehicles meeting

The length of a selected road section was measured. The time required for a vehicle to traverse this length without meeting vehicles traveling in the opposite direction was recorded. More than 10 vehicles were sampled, and the average velocity without vehicles meeting was obtained. A stopwatch was used to record the time required for the vehicle to travel from one end of the chosen section to the other end. Thus, the recorded time was the time required for the vehicle to travel without meeting an opposing vehicle on the given road section.

### Data extraction from the videos

Because cars are the most common type of vehicle in cities, we surveyed the meetings of cars. A car typically has a width of 1.5–2.0 m and a length of 4–5 m. The velocities of the vehicles during a meeting were calculated at each second, and the relationships between road width and vehicle velocity were analyzed.

From the videos, the distance that the vehicle traveled in each second was calculated. This information was used to determine the instantaneous velocity of the vehicle at each second. The velocities at the time of the meeting of the vehicles were then determined. The distance measurements were obtained using software tools on the map website http://map.qq.com/, which was used to identify each road of the city, the buildings along the street, and the distances between any two points.

## Results

**Fig 2:**
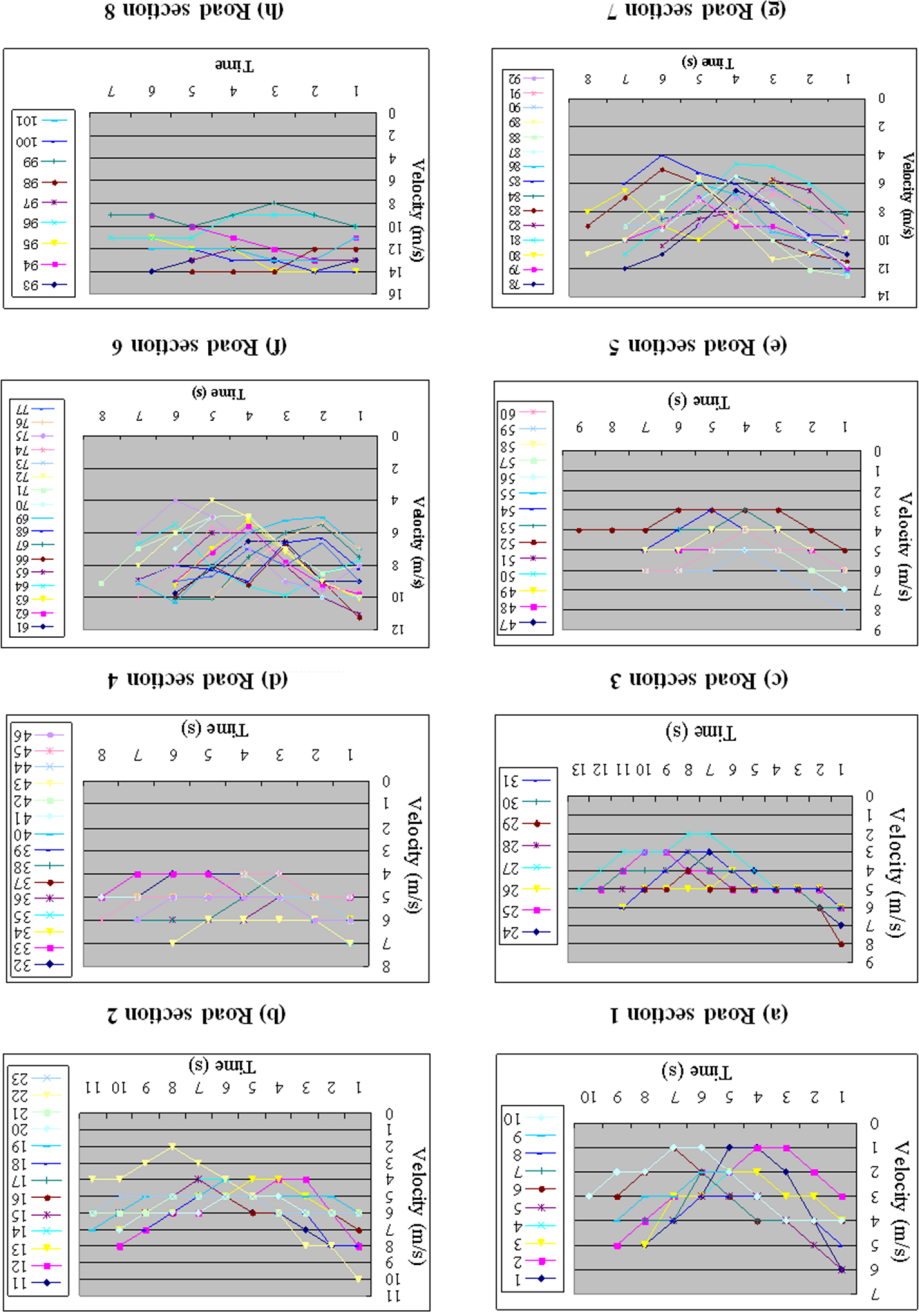
Velocity-time relationship of vehicles on each road section.

The velocities of the meeting vehicles at each second on each surveyed road section are listed in Tables 2–9 and graphed in Fig 2, in which the underlined values are the velocities at the moment of the meeting.

**Table 2:** Velocities of the meeting vehicles at each second on road section 1 (units: m/s).

Vehicle No.	1 s	2 s	3 s	4 s	5 s	6 s	7 s	8 s	9 s	10 s	11 s	12 s	13 s	14 s
1	6	4	***2***	***1***	***1***	3	4	5						
2	3	2	***1***	***1***	***2***	2	3	4	5					
3	4	3	***3***	***2***	***2***	3	3	5						
4	6	5	4	***3***	***2***	3	4							
5	6	5	4	***3***	***3***	3	4							
6	4	4	4	4	3	***2***	***1***	2	3					
7	4	4	4	4	***3***	***2***	4	4						
8	5	4	4	***3***	***3***	3	4							
9	4	4	4	3	***2***	***2***	***3***	3	4					
10	4	4	4	3	2	***1***	***1***	***2***	2	3				

**Table 3:** Velocities of the meeting vehicles at each second on road section 2 (units: m/s).

Vehicle No.	1 s	2 s	3 s	4 s	5 s	6 s	7 s	8 s	9 s	10 s	11 s
11	8	7	7	6	6	***5***	***5***	6	6	7	
12	8	6	***4***	***4***	5	5	6	6	7	8	
13	6	6	5	***4***	***4***	5	5	6	6	7	
14	6	6	6	6	5	***4***	***4***	5	6	6	
15	6	6	6	6	5	***5***	***4***	5	6	6	
16	7	6	6	6	6	***5***	***5***	6	6	6	
17	6	6	6	6	***5***	***4***	5	5	6	6	
18	7	7	6	6	***5***	***5***	5	6	7		
19	6	5	5	5	5	***4***	***5***	5	5	6	7
20	6	6	6	5	***5***	***5***	6	6	6	7	
21	6	6	6	6	5	***5***	***5***	5	6	6	6
22	7	7	7	6	5	4	***3***	***2***	***3***	4	4
23	6	6	6	6	5	5	***5***	***5***	5	5	6

**Table 4:** Velocities of the meeting vehicles at each second on road section 3 (units: m/s).

Vehicle No.	1 s	2 s	3 s	4 s	5 s	6 s	7 s	8 s	9 s	10 s	11 s	12 s	13 s
24	7	6	5	5	4	***4***	***3***	4	5	5	6		
25	6	5	5	5	5	5	4	***4***	***3***	***3***	4	5	
26	6	5	5	5	***5***	***4***	5	5	5	5	6		
27	6	5	5	5	4	***3***	***2***	***2***	3	3	3	4	5
28	6	5	5	5	5	5	***4***	***3***	4	5	5		
29	8	6	5	5	5	5	***5***	***4***	5	5			
30	6	6	5	5	5	4	***4***	***3***	4	4	4	5	
31	6	5	5	5	5	4	***3***	***3***	4	5	6		

**Table 5:** Velocities of the meeting vehicles at each second on road section 4 (units: m/s).

Vehicle No.	1 s	2 s	3 s	4 s	5 s	6 s	7 s	8 s
32	5	5	5	***4***	***4***	4	5	5
33	5	5	5	5	***4***	***4***	4	5
34	6	5	5	***5***	5	5	5	
35	5	5	***5***	6	6	6		
36	6	6	***5***	6	6	6		
37	6	5	5	***5***	5	5	6	
38	6	5	***4***	5	6	6	6	
39	6	5	5	***5***	5	5	6	
40	7	6	***5***	***4***	5	5	6	
41	5	5	***4***	***4***	5	5	5	5
42	6	5	***5***	***4***	5	5	5	
43	7	6	***6***	6	6	7		
44	6	5	5	***5***	5	5	6	
45	5	5	***4***	***4***	5	5	5	6
46	6	6	5	***5***	5	5	6	

**Table 6:** Velocities of the meeting vehicles at each second on road section 5 (units: m/s).

Vehicle No.	1 s	2 s	3 s	4 s	5 s	6 s	7 s	8 s	9 s
47	6	5	5	***4***	4	5			
48	6	5	5	***4***	5	5			
49	6	5	4	***4***	4	5	5		
50	6	5	4	4	***4***	4	5		
51	6	5	4	***4***	4	5	5		
52	5	4	3	***3***	***3***	3	4	4	4
53	6	5	4	***3***	***4***	4	5		
54	6	5	4	***4***	***3***	4	5		
55	7	6	5	***4***	5	6			
56	7	6	5	***5***	5	6			
57	6	6	5	***4***	5	6	6		
58	6	5	4	***4***	4	5	5		
59	8	7	6	***5***	6	6			
60	6	5	5	***4***	5	6	6		

**Table 7:** Velocities of the meeting vehicles at each second on road section 6 (units: m/s).

Vehicle No.	1 s	2 s	3 s	4 s	5 s	6 s	7 s	8 s
61	9	9	***7***	7	8	10		
62	10	9	8	***6***	7	9		
63	10	9	7	***5***	7	9		
64	8	9	10	9	8	***6***	7	
65	11	10	8	***6***	6	8	9	
66	11	9	***7***	9	8	10		
67	8	***6***	***6***	8	10	10		
68	8	6	***7***	9	8	8		
69	7	***5***	***5***	6	8	10	9	
70	10	10	9	7	***5***	7		
71	8	9	7	***5***	***5***	6	7	9
72	10	9	7	***5***	***4***	6	8	
73	10	10	9	7	***6***	8	9	
74	10	9	8	6	***6***	8	10	
75	8	10	9	7	***5***	***4***	6	
76	7	***5***	6	8	10			
77	9	***7***	8	7	9	9		

**Table 8:** Velocities of the meeting vehicles at each second on road section 7 (units: m/s).

Vehicle No.	1 s	2 s	3 s	4 s	5 s	6 s	7 s	8 s
78	11	10	8	***7***	9	11	12	
79	12	10	9	9	***7***	9	10	
80	10	8	***6***	8	10	9	***7***	8
81	8	6	***5***	***5***	7	9	11	
82	9	7	***6***	8	9	10		
83	12	11	10	8	***6***	***5***	7	9
84	8	8	***6***	6	8	9		
85	10	10	8	6	***5***	***4***	6	
86	12	10	9	7	***6***	8	10	
87	12	10	8	***6***	7	9		
88	13	12	10	8	***6***	7	9	
89	10	11	11	9	***6***	8	10	11
90	13	11	10	8	***7***	9	9	
91	9	7	***7***	8	7	9		
92	10	8	***6***	7	9			

**Table 9:** Velocities of the meeting vehicles at each second on road section 8 (units: m/s).

Vehicle No.	1 s	2 s	3 s	4 s	5 s	6 s	7 s
93	13	14	***13***	12	13	14	
94	11	13	12	***11***	10	9	
95	14	14	14	***12***	12	11	
96	10	9	***9***	9	11	11	11
97	13	13	13	***12***	13		
98	12	12	***14***	14	14		
99	10	9	8	9	***10***	9	9
100	14	14	***13***	13	12		
101	11	13	13	***12***	12	12	

The average velocities without vehicles meeting on road sections 1–8 were obtained by measuring the times required for the vehicles to travel the chosen road sections without meeting an opposing vehicle. These average velocities were 5.676, 6.723, 6.083, 6.162, 6.315, 10.134, 11.822, and 13.116 m/s for sections 1, 2, 3, 4, 5, 6, 7, and 8, respectively. It can be observed from Table 2 that because of the narrow road surface, the impedance caused by vehicles meeting is large (i.e., there is a large decrease in velocity when meeting). Comparing the data in Table 3 to the data in Table 2, the impedance caused by vehicles meeting is lower and the changes in velocity caused by meeting are clearly less on road section 2 than on road section 1. Comparing the data in Table 4 to the data in Table 3, there is no significant difference, though the road is more than 0.2 m wider in road section 3. Table 5 shows that when the road width was 5.30 m, the impedance caused by vehicles meeting decreased to a low level and the vehicle meetings lasted only 1–2 s. From Table 7, where the road width was 6.50 m, the average velocity was substantially higher; in particular, the velocity without vehicles meeting increased to more than 10 m/s, and the meeting speed also clearly increased. Comparing the data in Table 7 to the data in Table 8, for which the road widths were nearly equal (6.50 and 6.60 m, respectively), the meeting speeds were similar even though the average velocity in Table 8 was considerably higher. This result may have occurred because of a low traffic volume on road section 7, which demonstrates that the road width determines the meeting speed. When the road width was 7.6 m, meeting opposing vehicles had little influence on the velocities of the vehicles.

## Discussion

Table 10 presents the average meeting speed during meetings of opposing vehicles and the velocity decrease with respect to the velocity without meeting on branch roads of various widths.

**Table 10:** Average meeting speed and decrease in velocity compared to that without meeting.

Road width (m)	4.56	4.82	5.02	5.30	5.62	6.50	6.60	7.60
Average velocity without meeting (m/s)	5.676	6.723	6.083	6.162	6.315	10.134	11.822	13.116
Average meeting speed (m/s)	2.1	4.5	3.5	4.6	4.0	5.8	6.1	11.8
Velocity decrease when meeting (m/s)	3.576	2.223	2.583	1.562	2.315	4.334	5.722	1.316

Table 10 indicates that when the road width is less than 5.62 m, the average velocity of the vehicles on the branch road remains at a low level (approximately 6 m/s). When the road width increases from 4.56 to 4.82 m, the meeting speed sharply increases from 2.1 to 4.5 m/s. The meeting speed when the road width is 5.02 m is slightly smaller than that when the road width is 4.82 m, perhaps because the average velocity on road section 3 (width = 4.82 m) is higher and there is a small difference between the road widths of 4.82 and 5.02 m. For road widths of 4.82, 5.02, 5.30, or 5.62 m and a low average velocity (approximately 6 m/s without vehicles meeting), the meeting speed remains constant at approximately 4 m/s. When the road width increases from 4.82 to 5.62 m, the meeting speed does not increase considerably; thus, a wide road surface is not necessary; widening the road would increase land use and costs. On branch roads for which the average velocity of the vehicles is low (approximately 6 m/s), the road width should be more than 4.8 m because the meeting speed exhibits a sharp increase when the road width increases from 4.56 to 4.82 m; thus, a road width of 4.82 m can be considered a key parameter.

When the road width is 6.50 m, the average velocity increases to greater than 10 m/s, and the meeting speed increases to 5.8 m/s. The width of road section 7 was 6.60 m (nearly the width of road section 6, which was 6.50 m), and the average velocity on road section 7 is 11.822 m/s, perhaps because the traffic volume on road section 7 is lower. The average velocity on road section 7 is 2 m/s more than that on road section 6, but the meeting speeds are rather similar (5.8 and 6.1 m/s, respectively). Therefore, the road width is the determining factor influencing the meeting speed of bidirectional traffic. The road must be widened to increase the meeting speed.

When the road width is 7.6 m, the average velocity decreases only slightly (by 1.316 m/s) during the meetings of opposing vehicles, and the meeting velocity is high (approximately 13 m/s).

**Fig 3:**
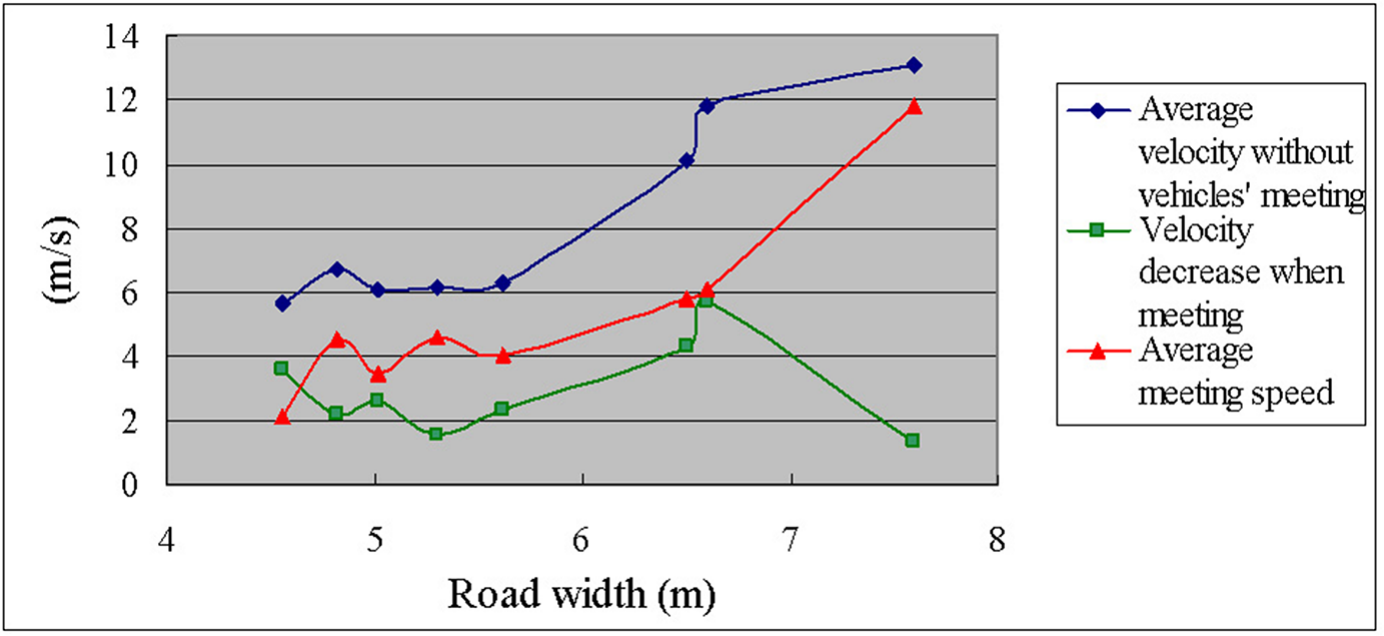
Average velocity without meeting and decrease in velocity when meeting versus the road width.

Fig 3 shows that when the road width increases from 5.62 to 6.5 m, the average velocity without meeting increases substantially, but the decrease in velocity when meeting also increases. Although the widths of road sections 6 and 7 are nearly equal, at 6.5 m and 6.6 m, respectively, the average velocity on road section 7 is 2 m/s higher than that on road section 6 because of a lower traffic volume on road section 7, and the decrease in velocity when meeting on road section 7 is significantly greater. Thus, a wider road surface is required to maintain higher speeds on the branch road. When the road width reaches 7.6 m, the velocity decrease that occurs during a meeting sharply decreases, though the average velocity is increasing.

**Fig 4:**
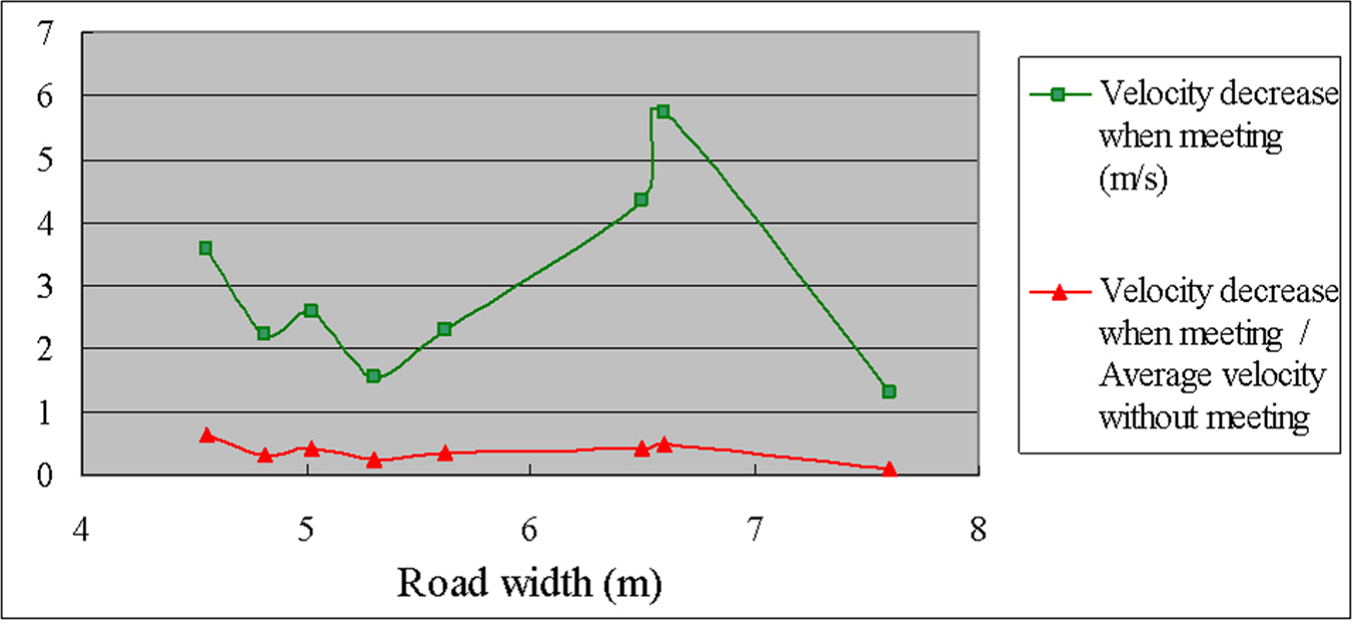
Velocity decrease when opposing vehicles meet and scaled velocity decrease versus road width.

As can be observed from Table 11 and Fig 4, the velocity decrease when opposing vehicles meet is significantly greater for a road width of 6.5 m than for a width of 5.62 m; however, the value of the scaled velocity decrease (velocity decrease when meeting/average velocity without meeting) is relatively low for road widths of 6.5 m and 6.6 m, and the maximum value occurs at the narrowest road width (4.56 m). Therefore, a road width of 4.56 m is obviously not adequate for the free flow of bidirectional traffic. When the road width increases from 4.56 m to 4.82 m, a relatively small increase, the value of the scaled velocity decrease sharply declines, so a road width larger than 4.82 m will significantly improve the meeting speed of bidirectional traffic. For a road width of 7.6 m, the value of the scaled velocity decrease is very small (only 0.1), which indicates that the speeds of opposing vehicles do not significantly change when they meet if the road is wide (7.6 m).

**Table 11:** Velocity decrease when opposing vehicles meet and velocity decrease scaled by average velocity without meeting for various road widths.

Road width (m)	4.56	4.82	5.02	5.30	5.62	6.50	6.60	7.60
Velocity decrease when meeting (m/s)	3.576	2.223	2.583	1.562	2.315	4.334	5.722	1.316
Velocity decrease when meeting / Average velocity without meeting	0.63	0.33	0.42	0.25	0.37	0.43	0.48	0.10

**Fig 5:**
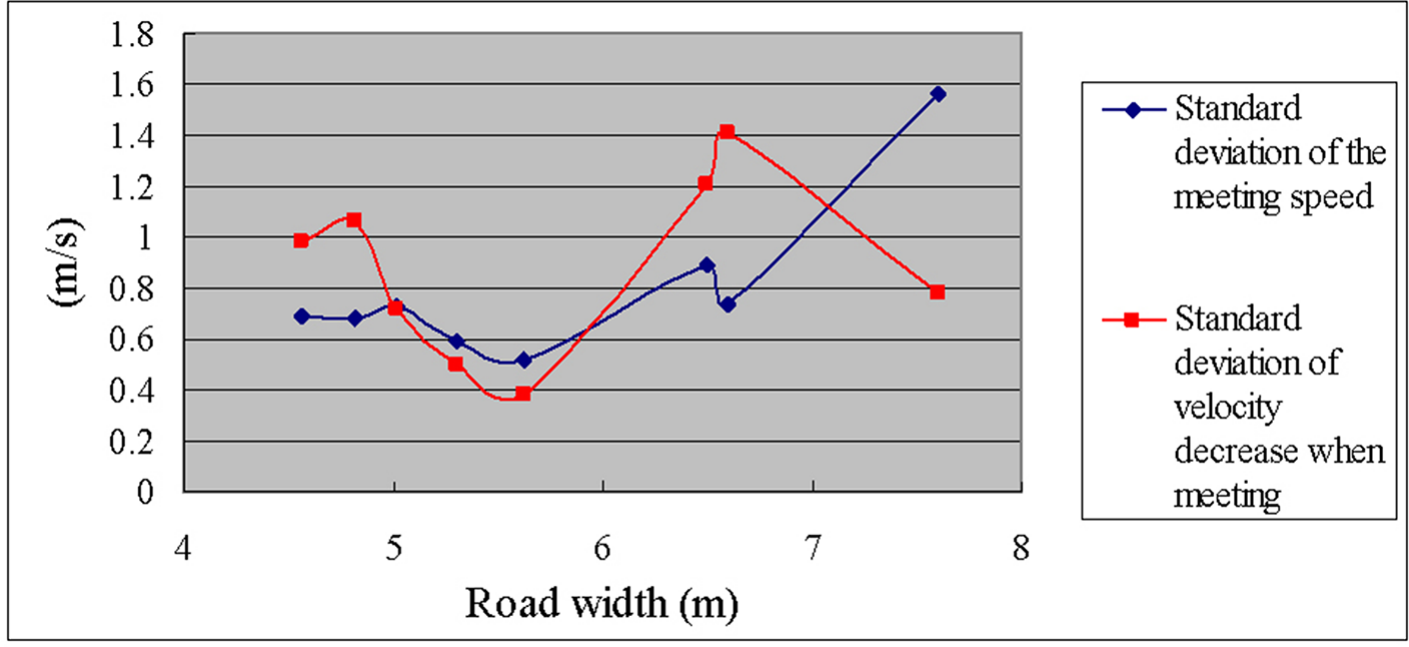
Standard deviations of the meeting speed and the velocity decrease when meeting versus road width.

**Fig 6:**
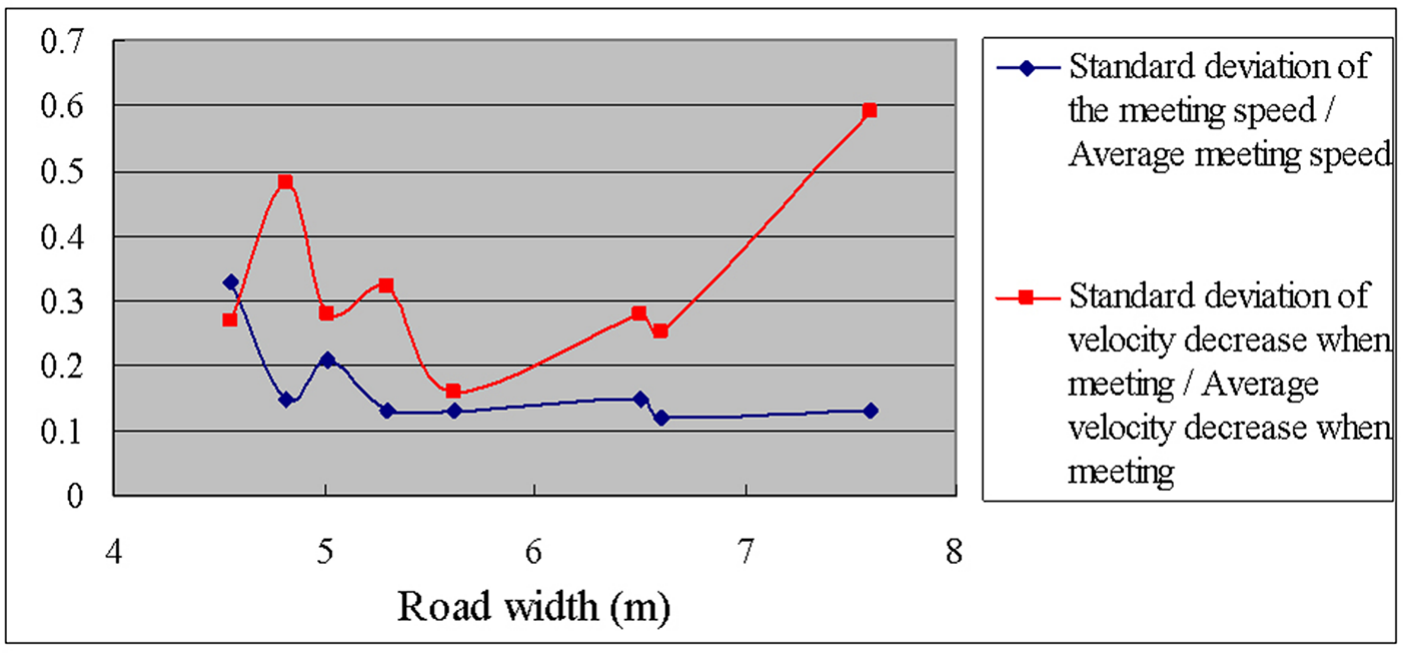
Coefficients of variation of the meeting speed and the velocity decrease when meeting versus road width.

In addition, we calculated the standard deviation and the coefficient of variation (standard deviation / average value) of the meeting speed and the velocity decrease when meeting to study the effect of the road width on the meeting speed; see Table 12 and Figs 5 and 6.

**Table 12:** Standard deviations and coefficients of variation of the meeting speed and the velocity decrease when meeting.

Road width (m)	4.56	4.82	5.02	5.30	5.62	6.50	6.60	7.60
Standard deviation of the meeting speed (m/s)	0.69	0.68	0.73	0.59	0.52	0.89	0.74	1.56
Standard deviation of the meeting speed / Average meeting speed	0.33	0.15	0.21	0.13	0.13	0.15	0.12	0.13
Standard deviation of velocity decrease when meeting (m/s)	0.98	1.06	0.72	0.50	0.38	1.21	1.41	0.78
Standard deviation of velocity decrease when meeting / Average velocity decrease when meeting	0.27	0.48	0.28	0.32	0.16	0.28	0.25	0.59

As can be observed from Table 12 and Figs 5 and 6, the standard deviation of the meeting speed is significantly higher only for a large road width (7.60 m), which demonstrates that the variation in speeds is greater when the road is wider (and the speed is generally high). However, the coefficient of variation (standard deviation of the meeting speed / average meeting speed) remains relatively low for a road width of 7.60 m. In contrast, the coefficient of variation is highest for a narrow road width (4.56 m), which demonstrates that the relative variation of the meeting speed is large when the road width is narrow.

Clear relationships between the standard deviation of the velocity decrease when meeting or the coefficient of variation and the road width were not observed.

The following conclusions can be drawn from the preceding analysis. (1) Branch roads require different road widths to prevent a large decrease in velocity when vehicles travelling in opposite directions meet: a higher average velocity corresponds to a wider road surface. When the average velocity exceeds 10 m/s, there is still a large decrease in velocity when meeting even if the road width reaches 6.6 m. Until the road width reaches 7.6 m, the velocity decrease when meeting decreases considerably. (2) Urban branch roads connect neighborhoods with major arteries and accommodate local traffic, and buildings line both sides of the road. Therefore, the branch roads cannot be particularly wide. Simply increasing the road width is not effective if the velocity on a branch road is high, but central dividers such as barriers can be used to separate the bidirectional traffic to increase the meeting velocity. Several studies have shown that central barriers can considerably increase the average velocity on a road [10, 11]. (3) The meeting speed is related to the road width. A wider road surface typically corresponds to a higher meeting speed (for a road width of 4.56 m, the meeting speed was 2.1 m/s, for a road width of approximately 5 m, the meeting speed was approximately 4 m/s, and for a road width of 6.5 m, the meeting speed was approximately 6 m/s). The meeting speed is also related to the average velocity on the road. If this average velocity is higher, the meeting speed is typically higher when the road width is fixed. For a road width of 4.82 m and an average velocity of 6.723 m/s, the meeting speed was 4.5 m/s. In contrast, for a road width of 5.02 m and an average velocity of 6.083 m/s, the meeting speed was 3.5 m/s. However, the road width has a greater effect on the meeting speed; e.g., for road widths of 6.5 and 6.6 m (i.e., nearly equal), the average velocities without meeting were 10.134 and 11.822 m/s, respectively (approximately 2 m/s difference), but the meeting speeds differed by only 0.3 m/s (5.8 and 6.1 m/s, respectively). (4) If the velocity on a branch road is not high (i.e., the average velocity without meeting is approximately 6 m/s), increasing the width of the road will increase the meeting velocity considerably (when the road width increases from 4.56 to 4.82 m, the meeting speed increases from 2.1 to 4.5 m/s). However, when the velocity is high (e.g., the average velocity without meeting is greater than 10 m/s), there is a large decrease (more than 4 m/s) in velocity when opposing vehicles meet if the road is 6.5 m wide. Therefore, simply increasing the road width is not effective when the velocity on a branch road is high, but central dividers such as barriers can be used to increase the meeting velocity.

Regarding urban branch roads, the criterion for lane width in Chinese cities is 3.5 m per lane [31, 32], the criterion in America is 3.3 m (3.0 m is also allowed for the low-speed lane, and even 2.7 m is allowed in residential areas) [33–35] and the criterion applied by Japan is 2.75–3.0 m width for one lane [36]. The survey performed in this study indicated that the requirement in China (3.5 m per lane) is high, allowing for a high average velocity and a high meeting speed on branch roads. If the velocity on a branch road is not high (i.e., the average velocity without meeting is approximately 6 m/s), then the decrease in velocity when opposing vehicles meet on such roads will be small. However, if the velocity is high (e.g., the average velocity without meeting is greater than 10 m/s), then central dividers such as barriers should be installed to increase the meeting velocity. However, this survey found that many branch roads do not satisfy the design criterion, resulting in a low average velocity and low meeting speeds. This result may be restricted by the actual conditions. The criteria in America are more flexible because different widths can be used depending on the average velocity and the location. Permitting lane widths of 3.0 m or 2.7 m for low-speed roads is consistent with the conclusions of this study. The lane widths in Japan are the lowest because land is less available there.
